# Association between patient continuity of care and physicians’ hypoglycaemic medication prescription trends

**DOI:** 10.1007/s10754-026-09410-4

**Published:** 2026-03-25

**Authors:** Joana Gomes-da-Costa, Sara R. Machado, Nuno Sousa Pereira

**Affiliations:** 1 Faculdade de Economia da Universidade do Porto, Porto, Portugal; 2 Centro de Economia e Finanças da Universidade do Porto, Porto, Portugal; 3 Brown University School of Public Health, Providence, USA; 4 LSE Health, London, UK

**Keywords:** Cardiovascular disease, Diabetes, Physician behaviour, Prescribing behaviour, Fragmentation of care, E-prescription, I10, I11

## Abstract

The general prevalence of chronic non-communicable diseases, such as diabetes mellitus is rapidly increasing while exacerbating the burden of disease on healthcare systems. Its management, as opposed to communicable diseases, is typically long term and requires ongoing healthcare interventions, such as dietary control and medication prescription, with associated costs. The prescription requires an interaction between patients and physicians, which may be sporadic or continuous, and can be used as a proxy measure for the strength of patient–doctor relationship. We hypothesize that fragmentation of care, across physician specialties and payers, plays a role on prescription behaviour, above and beyond for patient and prescription characteristics. A panel of patients’ prescriptions events with the universe of all prescriptions and dispensing in Portugal from January 2015 to October 2019 (*N* = 791.467) provided by Serviços Partilhados do Ministério da Saúde, EPE was considered. We measured the association between care fragmentation of care and prescription behavior of antihyperglycaemic medication using negative binomial regression models. Results suggest that Specialists play a secondary role on the prescription of DPP-4i and SGLT2i, prescribing 12.3 and 4.3% less respectively, while playing a central role on the prescription of GLP-1, in comparison with GPs. Fragmentation of care also plays a part on prescription trends, i.e., physicians with higher of continuity of care present higher rates of prescription of approximately 5.9% for DPP-4i, 6.5% for SGLT2i and 39.6% for GLP-1. The comparison of prescription trends amongst public and private payers suggests that public payers have lower rates of prescriptions (DPP-4i: 9.6%; SGLT2i: 7.2%; GLP-1: 85.6%). We find important differences in prescription patterns between specialists and primary care physicians. Higher continuity of care is associated with increased prescription frequency. Finally, public payers are associated with lower prescription rates. Physician specialty, payer, and care fragmentation all interact in the prescription patterns of antihyperglycaemic medication.

## Introduction

The general prevalence of chronic non-communicable diseases (NCDs), such as diabetes mellitus (DM) is rapidly increasing. Besides increasing mortality, the earlier development of NCDs have exacerbated the burden of disease on healthcare systems (Chan et al., 2021; Chen et al., 2013; Beadles et al., 2014).

As opposed to communicable diseases, the management for NCDs is typically long term and requires ongoing healthcare interventions, such as dietary control and medication prescription.

Medication prescription involves an interaction between patients and physicians which may be sporadic or continuous. While the first considers a fragmentation of care from multiple providers which is usually associated with duplication of services, medications, diagnostic tests, and procedures (Beadles et al., 2014), the second considers the repeated and coordinated contact between an individual patient and a doctor which can be used as a proxy measure for the strength of patient–doctor relationships (Waibel et al,. 2016, Pereira Gray et al., 2018).

Based on the framework for integrated people-centered health services, the World Health Organization recommends the practice of continuity of care to optimize the management on NCDs such as diabetes (Chan et al., 2021) and improve information sharing, alongside with longitudinal and interpersonal relationships between patients and their physicians (Chen et al., 2013). Relational continuity refers to “a therapeutic relationship between a patient and one or more providers that spans various healthcare events and results in accumulated knowledge of the patient and care consistent with the patient’s needs” (Chan et al., 2021). As providers gain more knowledge about the patient (Chan et al., 2021), they can tailor medical advice in subsequent consultations. Higher levels of continuity of care are usually associated with earlier diagnosis of a condition, reduced mortality, fewer hospitalizations, lower healthcare expenses, improved medication compliance, and higher patient satisfaction and quality of life (Chan et al., 2021, Chen et al., 2013, Beadles et al., 2014, Pereira Gray et al., 2018, Dossa et al. 2017).

On the contrary, fragmented care, or care that is insufficiently coordinated between providers, might be harmful to patients due to the duplication of diagnostic tests, inappropriate polypharmacy and conflicting care plans (Waibel et al., 2016).

A significant part of the costs associated with antidiabetic medicines may be attributed to the use of relatively recent pharmacologic agents in Type-2 Diabetes Mellitus (T2DM), such as glucagon-like peptide-1 (GLP-1) receptor agonists, dipeptidyl peptidase 4 inhibitors (DPP-4i) and sodium/glucose cotransporter 2 inhibitors (SGLT2i) (Emery et al., 2020). These three pharmacotherapeutic groups present different degrees of maturation regarding market positioning. This factor associated to its novelty and recent market introduction may lead to differences in prescription among physicians. This is an important matter due to variances among physician specialties, payers and the source of interaction between the patient and the provider.

This research paper considers the effect of fragmentation of care on prescription behaviour as well as it considers the effect of physician’s specialty (GPs vs. Specialists) and differences between public and private payer in the context of the Portuguese NHS, while controlling for patient and prescription characteristics.

For this purpose, we consider a panel of patients’ prescriptions events with the universe of all prescriptions and dispensing in Portugal from January 2015 to October 2019 provided by Serviços Partilhados do Ministério da Saúde, EPE (SPMS, EPE). The individual prescription data is matched with individual, physician, prescription drug, pharmacy and geographical characteristics enabling controlling for a broad range of cofounders.

Focusing our attention into the three pharmacotherapeutic groups of interest, we end up with 8,763 physicians prescribing 70,075 prescriptions containing Dipeptidyl peptidase 4 inhibitors (DPP-4i) to 10,008 patients; 6,311 physicians prescribing 38,075 prescriptions containing Sodium-glucose Cotransporter-2 inhibitors (SGLT2i) to 8,224 patients; and 2,005 physicians prescribing 10,156 prescriptions containing Glucagon-like Peptide-1 Receptor Agonists (GLP-1) to 2,714 patients.

We used graphical representations to study the prescription trend of these three pharmacotherapeutic groups followed by a Negative Binomial regression approach as our dependent variable is a count variable.

Most research has been focusing fragmentation of care, showing the benefits of higher levels of continuity. This study adds to the literature by considering the role of physician specialty on the continuity of care as well as the effect of public and private payer. To the best of our knowledge, this is the first study to estimate the impact of the physician work location on pharmaceutical prescription while accounting for prescription and patient characteristics.

## Methods

### Data

Prescriptions are an observable output that emerges from the patient-provider relationship. They reflect the supply side of health provided by physicians and the demand for treatment required by a patient.

We use a large longitudinal matched physician-prescription-patient dataset containing e-prescriptions collected by Serviços Partilhados do Ministério da Saúde, EPE (SPMS, EPE) between January 2015 and October 2019, covering all regions in Portugal.

Our data includes all payer – public and private sector – monthly prescription of the pharmacological class A10 - Drugs Used in Diabetes: A10B (Blood Glucose Lowering Drugs, excl. Insulins).

Prescriptions were selected if: (i) the patients were 18 years or older and (ii) if they had been prescribed with anti-diabetic pharmaceuticals within the selected time range.

SPMS, EPE provided anonymized information at the prescription level (id number, prescription date, dispensing date, cost of drug for the NHS, price supported by the patient, number of pills, pharmaceutical form, number of packages, dosage, active ingredient, and respective codes (CNPEM and national drug code) and posology). We also had access to information on the patient (age, gender, healthcare insurance, geographical location, health insurance), healthcare provider (medical specialty, workplace, type of care – hospital vs. primary care) and pharmacy where the prescription was dispensed (geographical location). Each line of observation corresponds to a single physician, which makes it our unit of observation.

### Outcome variables

**Fig. 1:**
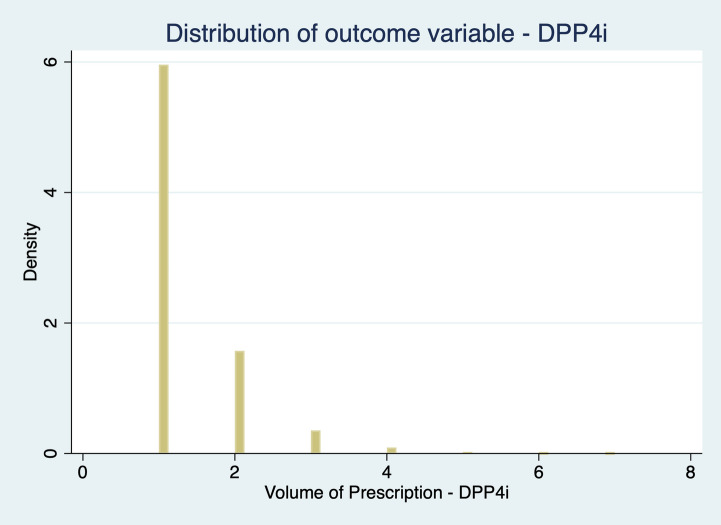
Distribution of outcome variable: DPP-4i

**Fig. 2:**
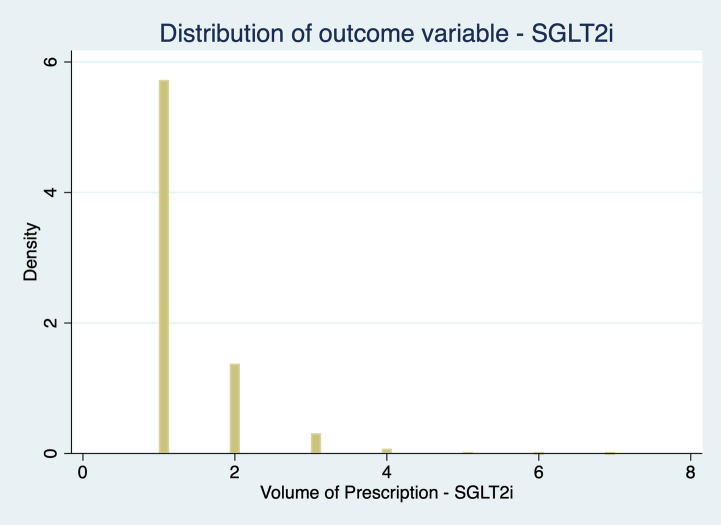
Distribution of outcome variable: SGLT2i

**Fig. 3:**
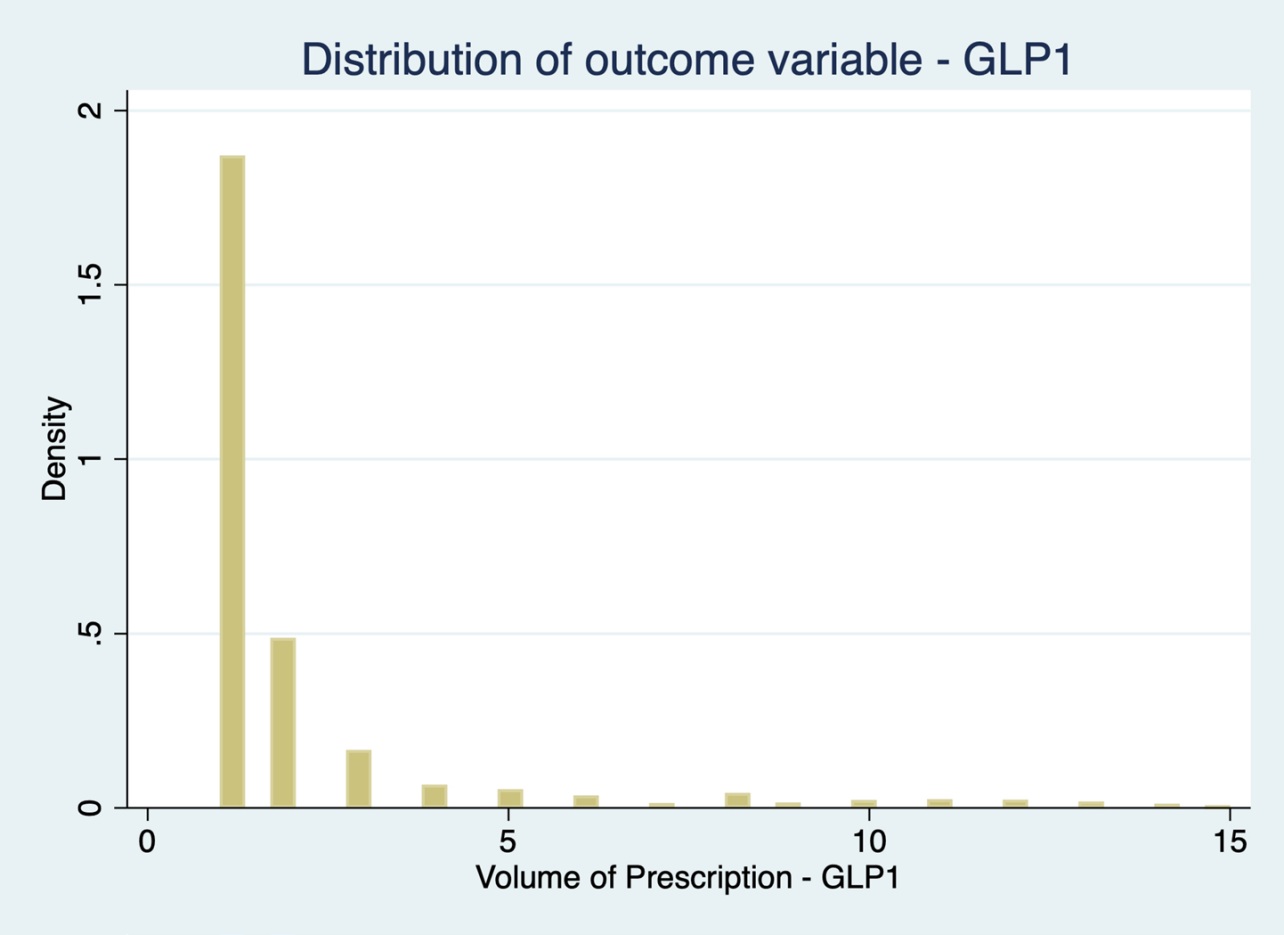
Distribution of outcome variable: GLP-1

The outcome variable – ${Prescription}_{in}$ – is defined as the volume of prescription by physician $\left(i\right)$ in month $\left(n\right)$. Figures 1, 2 and 3 represent the outcome variable distribution.

As our dependent variable is a count variable, i.e., a positive integer whose distribution is skewed to the left, we consider the Negative Binomial Model (NBM) the most appropriate choice (Cameron & Trivedi 1998) to estimate Eq. (1). We could also consider adopting a Poisson model, but our data suffer from two departures from the Poisson assumption. First, our data deals with “occurrence dependence”, i.e., the chances of a physician prescribing recent hypoglycaemic agents’ tomorrow are higher for physicians who prescribe them today (Winkelmann, 2015, Winkelmann & Zimmermann, 1995). Second, it also has into consideration “unobserved heterogeneity”, i.e., physicians have different prescription rates, with some prescribing more than others, due to different unobserved factors. Both occurrence dependence and unobserved heterogeneity invalidate the assumptions underlying the Poisson model (Winkelmann, 2015). Unobserved heterogeneity leads to “overdispersion”: in the conditional model for y as a function of x, the variance increases over-proportionally with the mean, i.e., the variance exceeds the mean (Winkelmann, 2015).

The NBM deals with both unobserved heterogeneity and occurrence dependence. Since our data are overdispersed – Table 1 – and NBM allows the variance to differ from the mean, this model yields better predictions of the outcome probabilities. This seems a pertinent solution to deal with unobserved heterogeneity in a count data.

**Table 1:** Dependent variable distribution

Prescription _in_	Mean	Variance	Standard Deviation	Minimum	Maximum
DPP-4i	1022.38	93472.86	305.73	74	1399
SGLT2i	731.07	141153.2	375.70	12	1495
GLP-1	113.76	3173.45	56.33	31	241

For robustness purposes, we will also be considering if the alpha parameter (dispersion parameter) reported for the negative binomial regression differs from zero, and evidence of overdispersion as well as the Pearson goodness-of-fit statistic which indicates overdispersion if degrees of freedom are much larger than 1. These results can be seen on Tables 5, 6, 7, 8, 9 and 10.

### Exposure variables

The explanatory variables included in the model are divided into prescription and patient's characteristics as described in Table 2.

**Table 2:** Description of variables

Variables	Description of variables
Prescription Characteristics	
$Type\;of\;physician$	= 1 if Specialist; 0 otherwise
$Month$	Month of Prescription
${Location}_{in}$	Proportion of public-payer (origin) prescriptions
${Renewable}_{in}$	Proportion of renewable prescriptions
${Inpatient}_{in}$	Proportion of inpatient prescriptions
$NHS$	Proportion of public insurance (NHS)
Patient Characteristics	
${Continuity}_{in}$	Proportion of single pair prescriptions
$Female$	= 1 for females; 0 otherwise
$Age$	Patient’s age
$Region$	= 1 if Alentejo; = 2 if Algarve; = 3 if Açores; = 4 if Centro; = 5 if Lisboa e Vale do Tejo; = 6 if Madeira; = 7 if Norte

Our approach considered six different scenarios:

1. No continuity of care, No prescription origin (public/private payer);
2. No continuity of care, Prescription origin (public/private payer);
3. Continuity of care, No prescription origin (public/private payer);
4. Continuity of care, Prescription origin (public/private payer);
5. Continuity of care, Prescription origin (public/private payer) adjusted for sociodemographic variables such as age and gender.
6. Continuity of care, Prescription origin (public/private payer) adjusted for sociodemographic variables such as age, gender and region.

The division of regressions undertaken in this analysis provides a more robust and nuanced comprehension of the research question. Specifically, the objective of this methodological approach is to assess the extent to which fragmentation of care is associated with potential modifications in prescription behavior across the three pharmacotherapeutic groups of interest — DPP-4i, GLP-1, and SGLT2i — when comparing prescribing patterns between general practitioners and specialists. Furthermore, this framework facilitates the examination of the mediating role of the payer (i.e., prescription origin), thereby enabling the identification of possible differences between public and private payers.

Scenario 4 was considered the preferred hypothesis to be tested.

### Analysis

The aim of this essay is to analyse to the relationship between fragmentation of care and physician prescription behavior in three diabetes-related pharmacotherapeutic groups (DPP-4i, GLP-1 and SGLT2i) across general practitioners and specialists.

We will also be considering the mediating role of payer (prescription origin) to study differences in physician prescription behaviour across public and private payers, estimated as.

1 $$\\\begin{array}{c}{Prescription}_{in}=\beta_0+\beta_1type+\\\beta_2{continuity}_{in}+\beta_3{location}_{in}+\\X_{in}+InteractionTerms+\epsilon_{in}\end{array}$$

We estimate this model using the three outcomes outlined above, per physician, per month. We adjust for ${X}_{in}$, including the proportion of renewable prescriptions, proportion of inpatient prescription, proportion of public insurance as well as patient’s age, gender and region. Interaction terms between the patient and the physician (continuity of care) as well as the payer’s origin (proportion of public-payer (origin) prescriptions are also included for some scenarios.

For this purpose, we consider a negative binomial model where the coefficients can be interpreted as follows: for a one-unit change in the predictor variable, the log of expected counts of the response variable changes by the respective regression coefficient, given the other predictor variables in the model are held constant.

## Results

### Physician-patient interactions across care settings

The total dataset contains 18,670 physicians, 84,750 patients, 792,467 e-prescriptions and 31 different oral hypoglycaemic agents.

To provide an overview of the sample, the descriptive statistics of the levels of continuity of care and the variables included in the model are reported in Tables 3 and 4, respectively.

**Table 3:** Descriptive statistics: levels of continuity of care

	Single Pair (Patient only sees 1 physician) *N* Pairs = 7,171			Non-Single Pair (Patient sees at least 2 physicians) *N* Pairs = 26,087		
	Public	Private	Public & Private	Public	Private	Public & Private
Prescriptions	4334	2837	-	22,637	3450	-
Patients (pair)	4284	2790	97	10,335	2441	174
Physicians (pair)	2703	1432	82	8282	1968	153
Patients	7171			11,656		
Physicians	3931			9734		
GP	2802 (71.27%)			8162 (83.85%)		
Endocrinologist	158 (4.22%)			173 (1.77%)		
Cardiologists	97 (2.46%)			151 (1.55%)		
Others	874 (22.22%)			1248 (12.83%)

**Table 4:** Descriptive statistics: physician, patient, prescription

	General Practitioners	Specialists
Physician	*N* = 8691	*N* = 2319
Continuity – Single Pair	1,437 (16.53%)	951 (41.01%)
Location – Public	7,551 (86.88%)	1,134 (48.90%)
Patient	*N* = 15,556	*N* = 3271
Age (mean)	66.95	59.29
Gender – Male	7,234 (46.50%)	1,286 (39.32%)
Gender – Female	8,322 (53.50%)	1,985 (60.68%)
Payer – NHS	14,428 (92.75%)	2,898 (88.60%)
Prescription	*N* = 101,358	*N* = 13,506
Hospital	18,600 (18.35%)	13,489 (99.87%)
Renewable	60,167 (59.36%)	5,439 (40.27%)

By single pair, we assume that there exists a continuity of care. This means that a specific patient sees only one physician during the observed period. By non-single pair, we assume that there exists a fragmentation of care and a specific patients can see more than one physician during the observed period.

Dividing the continuity of care among GPs and Specialists, we verify that: (i) prescriptions are more frequent among the public sector and (ii) physician-patient pairs are also more frequent among the public sector whether in continuity or fragmentation of care. Considering physician distribution, we’ve decided to divide them between GPs, Endocrinologists, Cardiologists, and other Specialties.

For the sake of simplicity, we are considering the first option. Our sample of single pairs contains 71.27% of GPs, 4.22% of Endocrinologists, 2.46% of Cardiologists and 22.22% for other Specialties. Complementary, our sample of non-single pairs contains 83.85% of GPs, 1.77% of Endocrinologists, 1.55% of Cardiologists and 12.83% for other Specialties.

Physicians are divided between General Practitioners (*N* = 8,691; 78.94%) and Specialists (*N* = 2,319; 21.06%). Approximately 17% of GPs are on a single pairing scheme, meaning that a specific patient is paired up with a specific GP. This patient does not see any other physician. Specialists on this position represent 41% of our sample.

General Practitioners are more frequently seen on a public setting (86.88%) while Specialists divide its presence between the public (48.90%) and private sector.

Patients recur to physicians in case of need to improve his health status. They can either look for a GP (*N* = 15,556; 82.63%) or a Specialist (*N* = 3,271; 17.37%). Patients associated to a GP are usually 6 years older than the ones associated to a Specialist (66.95 vs. 59.29 years older). Men (46.50%) tend to see a GP more often while women (60.68%) are usually more associated to Specialists. The Portuguese NHS is responsible for paying 92.75% of the patient’s interactions with the GP while paying 88.60% patient’s interactions with a Specialist.

Prescriptions are the output that comes out of a physician-patient interaction. General Practitioners are responsible for 88.24% (*N* = 101,358) of all prescriptions while Specialists are responsible for 11.76% (*N* = 13506).

Prescriptions from GPs mainly have a primary care origin, with 18.35% coming from an hospital setting. Prescriptions from specialists are majorly associated to a hospital setting with a total of 99.87%.

General Practitioners are responsible for 59.36% of renewable prescriptions while specialists are responsible for 40.27% of renewable prescriptions.

### DPP-4i, SGLT2i, GLP-1 prescription patterns

Focusing our attention into the three pharmacotherapeutic groups of interest, we end up with 8,763 physicians prescribing 70,075 prescriptions containing Dipeptidyl peptidase 4 inhibitors (DPP-4i) to 10,008 patients; 6,311 physicians prescribing 38,075 prescriptions containing Sodium-glucose Cotransporter-2 inhibitors (SGLT2i) to 8,224 patients; and 2,005 physicians prescribing 10,156 prescriptions containing Glucagon-like Peptide-1 Receptor Agonists (GLP-1) to 2,714 patients.

**Fig. 4:**
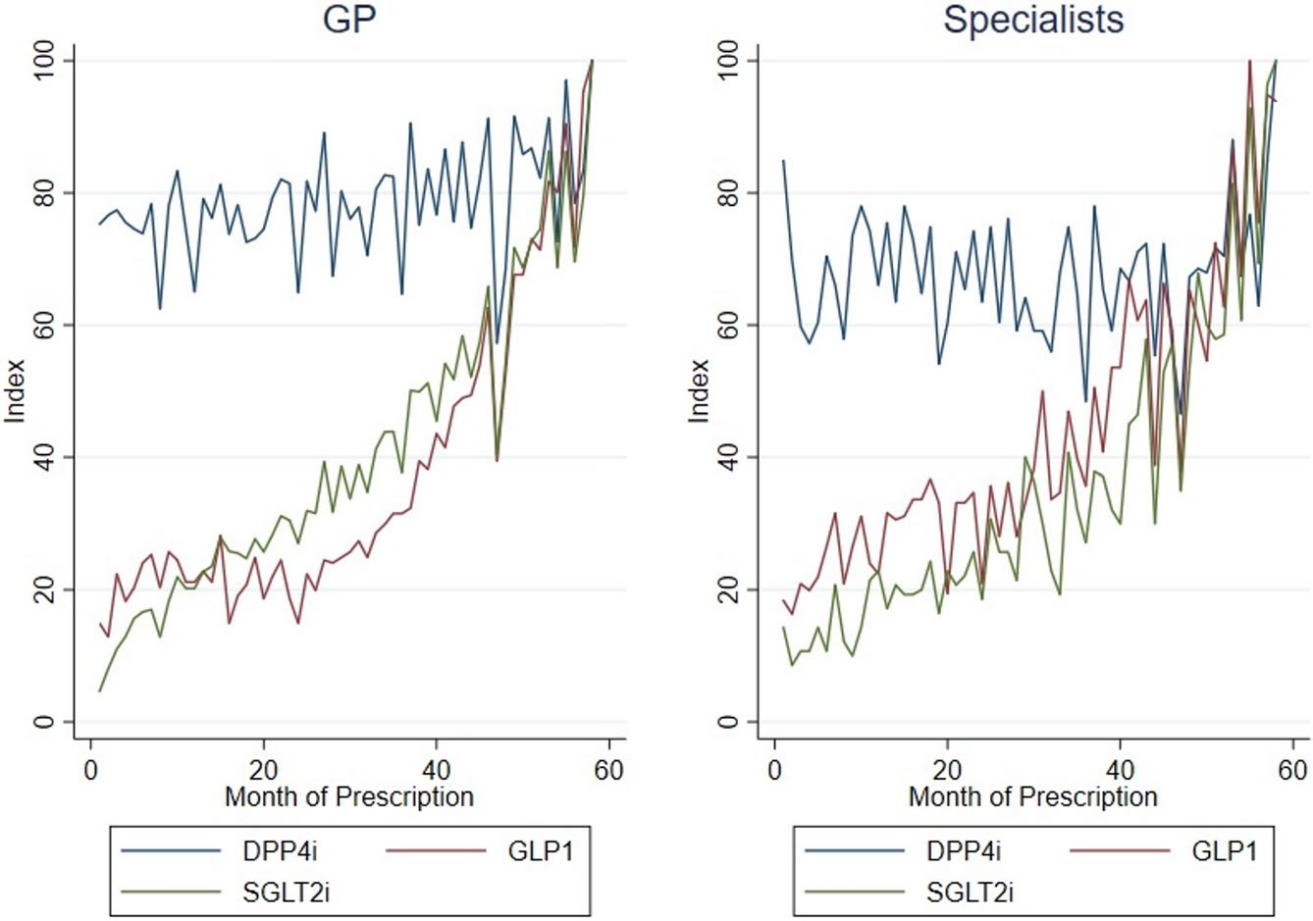
Trends in prescription of DPP-4i/SGLT2i/GLP-1: GP vs. Specialists

Figure 4 presents the trends in prescription of DPP-4i, SGLT2i and GLP-1 between GPs and Specialists, measured as an index using the maximum volume of prescription for each one of the groups as reference, as we are interested in rates of prescription.

The trends in prescriptions containing DPP-4i presents a more stable behaviour regardless of the physician specialty – GPs vs. Specialists. Although the prescribing pattern seems similar, the absolute volume of prescription with a GP-origin is 10 times higher than the volume of prescription with a Specialist-origin (mean of 1100.17 with GPs vs. 108.02 with Specialists). In both cases, DPP-4i seems the most popular option among GPs and Specialists.

As for SGLT2i and GLP-1, it is presented an increasing pattern throughout the observed period whether we consider prescriptions from a GP or from a Specialist.

Although prescription patterns may differ from GPs and Specialists, the final months of the observation period show that GLP-1 and SGLT2i present a similar prescription pattern in comparison with DPP-4i, whether they stay in similar levels (GPs) or whether they surpass DPP-4i (Specialists).

Patients can be paired up with GPs or Specialists. They can also be associated with the same physician during the observed period thus becoming a single patient-physician pair or they can choose to switch between physicians becoming a non-single patient-physician pair introducing care fragmentation. For the sake of simplification, non-single pairs will only allow patients to switch from one GP to another and from one Specialist to another.

**Fig. 5:**
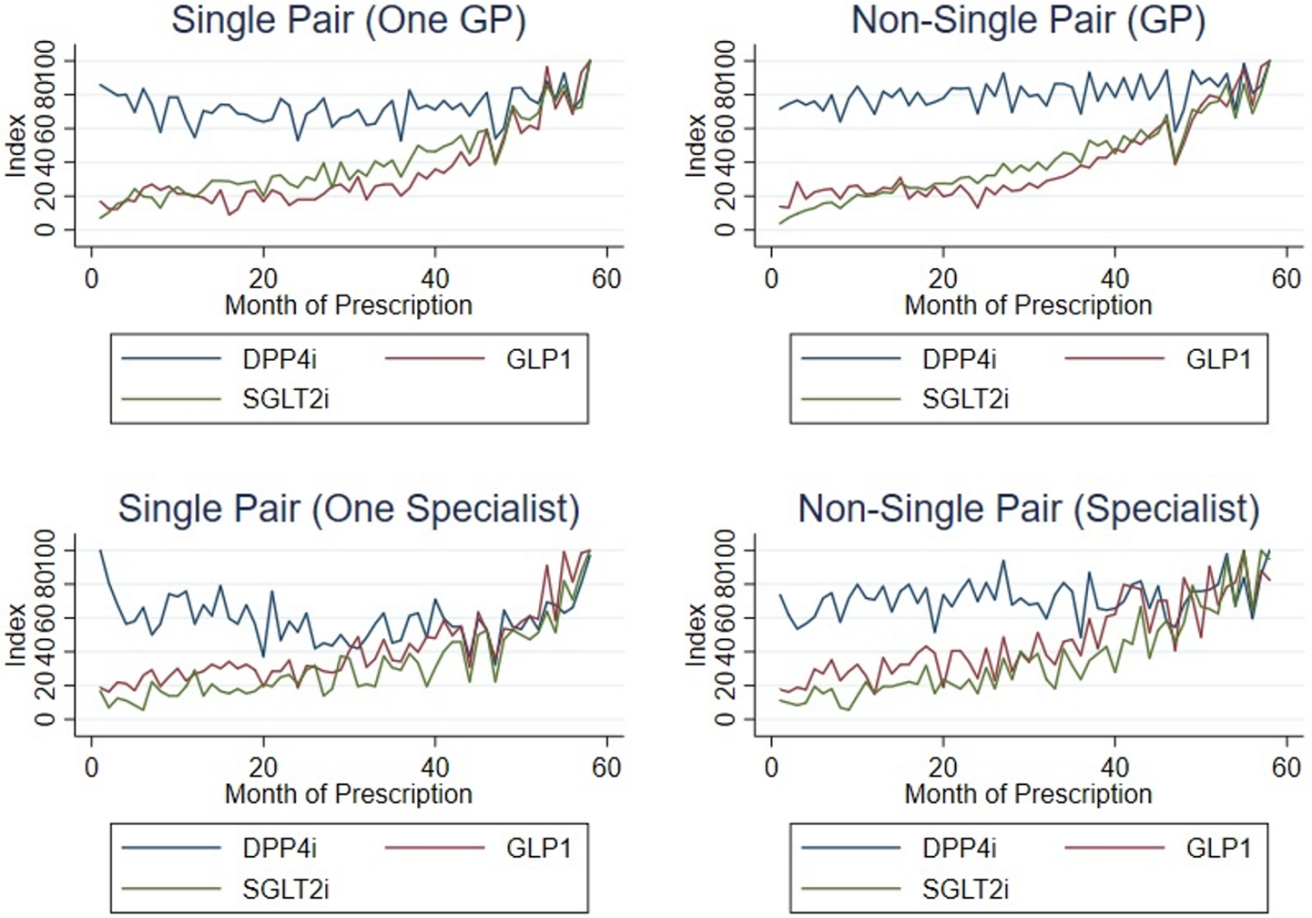
Trends in prescription of DPP-4i/SGLT2i/GLP-1: single pair vs. non-single pair

Figure 5 presents the trends in prescription of DPP-4i, SGLT2i and GLP-1 between single pairs and non-single pairs using an index using the maximum volume of prescription for each one of the group as reference, as we are interested in verifying whether fragmentation of care modifies the previous prescription trends showed for GPs and Specialists.

General Practitioners, in single and non-single pairs, show a clear preference for DPP-4i prescription which presents a stable behaviour over time for each one of the pairing schemes. These physicians also prescribe SGLT2i and GLP-1 at an increasing rate during the observed period. By the beginning of 2019, the three pharmacotherapeutic groups are prescribed at a similar rate producing a corresponding behaviour between them.

Prescription among Specialists, in single and non-single pairs, also present a preference towards DPP-4i, which keeps showing a stable pattern of prescription. These physicians also prescribe GLP-1 and SGLT2i, representing a second and third option of prescription, respectively, after the prescription of DPP-4i.

Similarly to what happens with GPs, these two pharmacotherapeutic groups also present an increasing trend of prescription during the observed period. By mid-2018, earlier than what happens with GPs, the three groups are prescribed at a similar rate producing a corresponding behaviour between them.

### Association between patient continuity mix and prescription rates, by physician specialty type

Tables 5, 6, 7, 8, 9 and 10 presents the differences between public and private origin on prescription behavior, while also considering physician specialty and fragmentation of care.

**Table 5:** Regression analysis for DPP-4i – Cases 1 to 3

Variables	Case 1 (a)	Case 2 (a)	Case 3 (a)	Case 3 (a) (d)
*type *(=1)	−0.141***	−0.121***	−0.143***	−0.133***
	(0.007)	(0.007)	(0.007)	(0.007)
*inpatient*_in_	0.033***	−0.052***	0.026***	0.025***
	(0.005)	(0.008)	(0.005)	(0.005)
*location*_in_		−0.104***		
		(0.007)		
*continuity*_in_			0.063***	0.066***
			(0.004)	(0.005)
*continuity*_in_#type				−0.028**
				(0.011)
*continuity*_in_* #location*_in_				
Constant	0.327***	0.427***	0.312***	0.311***
	(0.004)	(0.008)	(0.004)	(0.004)
Alpha	−26.30	−26.30	−26.30	−26.30
	(0)	(0)	(0)	(0)
Goodness-of-fit (for Poisson regression)	22059.11	21975.72	21907.48	21904.85
Observations	70,075	70,075	70,075	70,075

Dependent Variable: Volume of prescription by physician (i) in month (n)

Standard errors in parentheses; *** *p* < 0.01, ** *p* < 0.05, * *p* < 0.1

(a). Regression includes control variables such as the proportion of renewable prescriptions, proportion of inpatient prescriptions and month of prescription.

(b). Regression includes control variables such as the proportion of renewable prescriptions, proportion of inpatient prescriptions, month of prescription, proportion of public insurance as well as patient’s age and gender.

(c). Regression includes all control variables included on *X*_in_

(d). Regression includes the interaction terms

**Table 6:** Regression analysis for DPP-4i – cases 4 to 6

Variables	Case 4 (a)	Case 4 (a) (d)	Case 5 (b)	Case 5 (b) (d)	Case 6 (c)	Case 6 (c) (d)
$type\;(=1)$	−0.123***	−0.121***	−0.120***	−0.116***	−0.099***	−0.095***
	**(0.007)**	(0.008)	(0.007)	(0.008)	(0.008)	(0.008)
${inpatient}_{in}$	−0.052***	−0.054***	−0.053***	−0.054***	−0.049***	−0.052***
	**(0.007)**	(0.008)	(0.008)	(0.008)	(0.009)	(0.009)
${location}_{in}$	−0.096***	−0.120***	−0.096***	−0.120***	−0.095***	−0.119***
	**(0.007)**	(0.008)	(0.007)	(0.008)	(0.008)	(0.009)
${continuity}_{in}$	0.059***	0.007	0.059***	0.008	0.054***	0.004
	**(0.004)**	(0.009)	(0.004)	(0.008)	(0.004)	(0.009)
${continuity}_{\mathrm{in}}\;\#type$		−0.004		−0.007		−0.008
		(0.011)		(0.0112)		(0.011)
${continuity}_{in}\;\#{location}_{in}$		0.070***		0.069***		0.067***
		(0.009)		(0.009)		(0.009)
$age$			2.95e-05	3.43e-05	−0.0002	−0.0002
			(0.0002)	(0.0002)	(0.0002)	(0.0002)
$gender$			−0.021***	−0.021***	−0.021***	−0.021***
			(0.004)	(0.004)	(0.004)	(0.004)
$Alentejo$					0.152***	0.152***
					(0.007)	(0.007)
$Algarve$					0.086***	0.086***
					(0.009)	(0.009)
$A\varsigma ores$					0.049***	0.052***
					(0.014)	(0.014)
$Centro$					0.087***	0.086***
					(0.005)	(0.005)
$Madeira$					0.056***	0.059***
					(0.014)	(0.014)
$Norte$					0.015***	0.015***
					(0.005)	(0.005)
Constant	0.406***	0.427***	0.397***	0.417***	0.344***	0.365***
	**(0.008)**	(0.009)	(0.015)	(0.016)	(0.016)	(0.016)
Alpha	**−26.30**	−26.30	−26.30	−26.30	−26.30	−26.30
	**(0)**	(0)	(0)	(0)	(0)	(0)
Goodness-of-fit (for Poisson regression)	**21838.41**	21800.79	21817.3	21779.65	21366.31	21333.3
Observations	**70**,**075**	70,075	70,075	70,075	70,075	70,075

Dependent Variable: Volume of prescription by physician (i) in month (n)

Standard errors in parentheses; *** *p* < 0.01, ** *p* < 0.05, * *p* < 0.1

(a). Regression includes control variables such as the proportion of renewable prescriptions, proportion of inpatient prescriptions and month of prescription.

(b). Regression includes control variables such as the proportion of renewable prescriptions, proportion of inpatient prescriptions, month of prescription, proportion of public insurance as well as patient’s age and gender.

(c). Regression includes all control variables included on *X*_in,_

(d). Regression includes the interaction terms

**Table 7:** Regression analysis for SGLT2i – Cases 1 to 3

Variables	Case 1 (a)	Case 2 (a)	Case 3 (a)	Case 3 (a) (d)
*type* (=1)	−0.027**	−0.035***	−0.036***	−0.068***
	(0.013)	(0.012)	(0.013)	(0.013)
*inpatient*_in_	0.051***	−0.001	0.044***	0.045***
	(0.008)	(0.010)	(0.007)	(0.008)
*location*_in_		−0.082***		
		(0.011)		
*continuity*_in_			0.068***	0.061***
			(0.005)	(0.006)
*continuity*_in_ #type				0.068***
				(0.019)
*continuity*_in_ #location_in_				
Constant	0.231***	0.309***	0.212***	0.214***
	(0.007)	(0.013)	(0.007)	(0.007)
Alpha	−51.28	−51.28	−51.28	−51.28
	(0)	(0)	(0)	(0)
Goodness-of-fit (for Poisson regression)	11209.63	11171.66	11130.45	11118.64
Observations	38,075	38,075	38,075	38,075

Dependent Variable: Volume of prescription by physician (i) in month (n)

Standard errors in parentheses; *** *p* < 0.01, ** *p* < 0.05, * *p* < 0.1

(a). Regression includes control variables such as the proportion of renewable prescriptions, proportion of inpatient prescriptions and month of prescription.

(b). Regression includes control variables such as the proportion of renewable prescriptions, proportion of inpatient prescriptions, month of prescription, proportion of public insurance as well as patient’s age and gender.

(c). Regression includes all control variables included on *X*_in_

(d). Regression includes the interaction terms

**Table 8:** Regression analysis for SGLT2i – Cases 4 to 6

Variables	Case 4 (a)	Case 4 (a) (d)	Case 5 (b)	Case 5 (b) (d)	Case 6 (c)	Case 6 (c) (d)
$type(=1)$	−0.043***	−0.060***	−0.039***	−0.064***	−0.022*	−0.045***
	**(0.012)**	(0.013)	(0.012)	(0.013)	(0.013)	(0.014)
${inpatient}_{in}$	**−0.001**	0.0004	−0.001	0.0006	−0.036**	−0.034**
	**(0.010)**	(0.010)	(0.010)	(0.010)	(0.014)	(0.014)
${location}_{in}$	−0.072***	−0.058***	−0.071***	−0.057***	−0.110***	−0.098***
	**(0.011)**	(0.013)	(0.011)	(0.013)	(0.013)	(0.014)
${continuity}_{in}$	0.065***	0.085***	0.067***	0.087***	0.063***	0.079***
	**(0.005)**	(0.014)	(0.005)	(0.014)	(0.005)	(0.014)
${continuity}_{in}\;\#type$		0.048**		0.049**		0.043**
		(0.020)		(0.020)		(0.020)
${continuity}_{in\;}\#{location}_{in}$		−0.033**		−0.033**		−0.026*
		(0.015)		(0.015)		(0.015)
$age$			0.001***	0.001***	0.001***	0.001***
			(0.0002)	(0.0002)	(0.0002)	(0.0002)
$gender$			−0.006	−0.006	−0.005	−0.004
			(0.005)	(0.005)	(0.005)	(0.005)
$Alentejo$					0.055***	0.054***
					(0.009)	(0.009)
$Algarve$					0.018	0.017
					(0.012)	(0.012)
$A\varsigma ores$					0.170***	0.166***
					(0.019)	(0.019)
$Centro$					0.067***	0.067***
					(0.007)	(0.007)
$Madeira$					−0.0002	−0.002
					(0.016)	(0.016)
$Norte$					0.059***	0.058***
					(0.006)	(0.007)
Constant	0.282***	0.270***	0.227***	0.214***	0.205***	0.195***
	**(0.013)**	(0.014)	(0.022)	(0.0220)	(0.023)	(0.024)
Alpha	**−51.28**	−51.28	−51.28	−51.28	−51.28	−51.28
	**(0)**	(0)	(0)	(0)	(0)	(0)
Goodness-of-fit (for Poisson regression)	**11102.16**	11089.69	11089.76	11076.72	10985.66	10976.62
Observations	**38**,**075**	38,075	38,075	38,075	38,075	38,075

Dependent Variable: Volume of prescription by physician (i) in month (n)

Standard errors in parentheses; *** *p* < 0.01, ** *p* < 0.05, * *p* < 0.1

(a). Regression includes control variables such as the proportion of renewable prescriptions, proportion of inpatient prescriptions and month of prescription.

(b). Regression includes control variables such as the proportion of renewable prescriptions, proportion of inpatient prescriptions, month of prescription, proportion of public insurance as well as patient’s age and gender.

(c). Regression includes all control variables included on *X*_in_

(d). Regression includes the interaction terms

**Table 9:** Regression analysis for GLP-1 – Cases 1 to 3

Variables	Case 1 (a)	Case 2 (a)	Case 3 (a)	Case 3 (a) (d)
$type\;(=1)$	0.373***	0.420***	0.337***	0.0517**
	(0.022)	(0.021)	(0.021)	(0.0213)
${inpatient}_{in}$	0.528***	−0.283***	0.360***	0.451***
	(0.017)	(0.023)	(0.018)	(0.017)
${location}_{in}$		−0.928***		
		(0.019)		
${continuity}_{in}$			0.483***	
			(0.016)	
${continuity}_{in\;}\#type$				0.465***
				(0.029)
$${continuity}_{in}\;\#{location}_{in}$$				
Constant	0.004	0.866***	−0.117***	−0.054**
	(0.026)	(0.029)	(0.027)	(0.026)
Alpha	−1.292***	−1.503***	−1.392***	−1.403***
	(0.032)	(0.032)	(0.032)	(0.032)
Goodness-of-fit (for Poisson regression)	19375.83	15649.92	17439.6	17125.12
Observations	10,156	10,156	10,156	10,156

Dependent Variable: Volume of prescription by physician (i) in month (n)

Standard errors in parentheses; *** *p* < 0.01, ** *p* < 0.05, * *p* < 0.1

(a). Regression includes control variables such as the proportion of renewable prescriptions, proportion of inpatient prescriptions and month of prescription.

(b). Regression includes control variables such as the proportion of renewable prescriptions, proportion of inpatient prescriptions, month of prescription, proportion of public insurance as well as patient’s age and gender.

(c). Regression includes all control variables included on *X*_in_

(d). Regression includes the interaction terms

**Table 10:** Regression analysis for SGLT2i – Cases 4 to 6

Variables	Case 4 (a)	Case 4 (a) (d)	Case 5 (b)	Case 5 (b) (d)	Case 6 (c)	Case 6 (c) (d)
$type\;(=1)$	0.389***	0.201***	0.391***	0.212***	0.396***	0.207***
	**(0.020)**	(0.022)	(0.019)	(0.023)	(0.020)	(0.024)
${inpatient}_{in}$	−0.360***	−0.215***	−0.369***	−0.229***	−0.329***	−0.174***
	**(0.024)**	(0.022)	(0.024)	(0.022)	(0.026)	(0.025)
${location}_{in}$	−0.856***	−0.633***	−0.848***	−0.633***	−0.794***	−0.576***
	**(0.019)**	(0.021)	(0.019)	(0.021)	(0.021)	(0.023)
${continuity}_{in}$	0.396***	0.360***	0.359***	0.329***	0.351***	0.307***
	**(0.016)**	(0.026)	(0.017)	(0.027)	(0.017)	(0.028)
${continuity}_{in}\;\#type$		0.297***		0.283***		0.291***
		(0.029)		(0.029)		(0.030)
${continuity}_{in\;}\#{location}_{in}$		−0.390***		−0.377***		−0.359***
		(0.027)		(0.027)		(0.028)
$age$			−0.003***	−0.002***	−0.002***	−0.002***
			(0.0007)	(0.0007)	(0.0007)	(0.001)
$gender$			−0.056***	−0.051***	−0.052***	−0.047**
			(0.019)	(0.019)	(0.019)	(0.019)
$Alentejo$					−0.036	−0.068**
					(0.034)	(0.034)
$Algarve$					−0.292***	−0.278***
					(0.031)	(0.031)
$A\varsigma ores$					0.0603	−0.036
					(0.045)	(0.047)
$Centro$					0.087***	0.075***
					(0.028)	(0.028)
$Madeira$					−0.089	−0.110*
					(0.061)	(0.059)
$Norte$					0.179***	0.172***
					(0.023)	(0.023)
Constant	0.701***	0.577***	0.651***	0.532***	0.470***	0.353***
	**(0.029)**	(0.030)	(0.053)	(0.056)	(0.057)	(0.058)
Alpha	−1.592***	−1.613***	−1.615***	−1.635***	−1.664***	−1.684***
	**(0.033)**	(0.034)	(0.034)	(0.034)	(0.034)	(0.035)
Goodness-of-fit (for Poisson regression)	**14504.46**	14125.08	14274.71	13926.48	13788.71	13456.9
Observations	**10**,**156**	10,156	10,156	10,156	10,156	10,156

Dependent Variable: Volume of prescription by physician (i) in month (n)

Standard errors in parentheses; *** *p* < 0.01, ** *p* < 0.05, * *p* < 0.1

(a). Regression includes control variables such as the proportion of renewable prescriptions, proportion of inpatient prescriptions and month of prescription.

(b). Regression includes control variables such as the proportion of renewable prescriptions, proportion of inpatient prescriptions, month of prescription, proportion of public insurance as well as patient’s age and gender.

(c). Regression includes all control variables included on *X*_in_

(d). Regression includes the interaction terms

Prescription practices related with DPP-4i, SGLT2i and GLP-1 can vary according to physician’s specialty. Our results suggest that Specialists play a secondary role on the prescription of DPP-4i and SGLT2i, while playing a central role on the prescription of GLP-1, in comparison with GPs. Specialists prescribe 9.5 to 14.3% (12.3%) and 2.7 to 6.8% (4.3%) less medicines from DPP-4i and SGLT2i, while prescribing more 5.2 to 42% (38.9%) more medicines from GLP-1.

Fragmentation of care can be considered as a factor that modifies these prescription behaviour. Results suggest that physicians with a higher proportion of single pairs (continuity scheme) present higher rates of prescription for the three pharmacotherapeutic groups previously considered. Physicians on single-pairing schemes prescribe approximately 5.9 to 6.3% (5.9%) more DPP-4i, 6.5 to 8.5% (6.5%) more SGLT2i and 30.7 to 48.3% (39.6%) more GLP-1.

Information available for each prescription also allows the comparison of prescription behavior among public and private payers. Results suggest that an increase on the proportion of prescriptions with a public origin, decrease the prescription of DPP-4i, SGLT2i and GLP-1.

This effect is particularly significant for GLP-1, where a higher proportion of public prescriptions by physician lead to 57.6 to 92.8% (85.6%) less prescriptions containing these medicines. Concerning DPP-4i and SGLT2i, a higher proportion of public-origin prescriptions is associated with less 9.5 to 12% (9.6%) and 5.7 to 11% (7.2%) less prescriptions, respectively.

The effect of continuity is estimated across all physicians. When interacting terms between continuity and physician specialty, we do not find coefficients with statistical significance for DPP-4i, however results suggest that the impact of continuity of care is amplified when the physician is a specialist, especially for SGLT2i and GLP-1. Specifically, for patients treated by specialists, each one-unit increase in continuity of care is associated with a 4.3 to 6.8% (SGLT2i) and 28.3 to 46.5% (GLP-1) higher expected count relative to patients treated by generalists, after adjusting for other covariates.

When interacting terms between continuity and payer type results differ. For DPP-4i, results suggest that the beneficial impact of continuity of care is amplified in populations with greater reliance on public payer prescriptions, i.e., as the proportion of public payer prescriptions increases, each one-unit increase in continuity of care is associated with a 7% higher expected count relative to contexts with lower public payer proportions, after adjusting for other covariates.

For SGLT2i and GLP-1 the results are the opposite, i.e., the beneficial impact of continuity of care is attenuated in populations with greater reliance on public payer prescriptions. Specifically, as the proportion of public payer prescriptions increases, each one-unit increase in continuity of care is associated with a 2.6 to 3.3% (SGLT2i) and 35.9 to 39% (GLP-1) lower expected count relative to contexts with fewer public payer prescriptions, after adjusting for other covariates.

We also estimated models allowing for age, gender and region heterogeneity.

Results for DPP-4i and SGLT2i suggest that an additional year is associated with higher expected prescription volume per month, while for GLP-1 age is associated with lower expected prescription volume per month.

Also, being female is associated with a lower expected monthly prescription volume, after adjusting for other covariates. Results also suggests a modest gender difference in prescribing patterns.

We also find regional differences in prescription behavior between Lisboa e Vale do Tejo and the rest of the country, with opposite effects between DPP-4i/SGLT2i and GLP-1.

To address potential indication bias, we conducted additional robustness checks examining whether disease severity was systematically associated with greater care fragmentation. Table 11 present the results.

**Table 11:** Robustness checks

Variables	Outcome variable: Number of physicians	Outcome variable: Number of specialties
Disease Severity	0.127***	0.005***
	(0.001)	(0.0003)
$age$	0.003***	−0.0006***
	(5.93e-05)	(2.50e-05)
$gender$	−0.0001	0.008***
	(0.003)	(0.0005)
$Alentejo$	−0.067***	−0.063***
	(0.003)	(0.001)
$Algarve$	0.055***	−0.052***
	(0.004)	(0.001)
$A\varsigma ores$	−0.011**	0.031***
	(0.005)	(0.003)
$Centro$	−0.107***	−0.057***
	(0.002)	(0.001)
$Madeira$	−0.343***	0.026***
	(0.005)	(0.003)
$Norte$	−0.089***	−0.055***
	(0.002)	(0.001)
Constant	0.75***	0.108***
	(0.005)	(0.02)
Observations	792,467	792,467

Dependent Variable: Volume of prescription by physician (i) in month (n)

Standard errors in parentheses; *** *p* < 0.01, ** *p* < 0.05, * *p* < 0.1

First, we estimated a negative binomial model with the number of physicians consulted as the dependent variable. The coefficient for disease severity was 0.127, implying that, holding other factors constant, a one-unit increase in severity is associated with a 14% increase in the expected number of physicians seen. Second, we estimated an analogous negative binomial model using the number of specialties visited as the outcome. The coefficient for disease severity was 0.0049, indicating only a 0.5% increase in the expected number of specialties consulted per unit increase in severity. Taken together, these results show that while more severe patients tend to see slightly more physicians, disease severity is not meaningfully associated with specialty-level fragmentation. Importantly, incorporating these measures of clinical need did not alter the magnitude or significance of our main findings.

## Discussion

Doctors behave differently. Differences on prescription patterns are an important output that can translate physician’s behaviour. The aim of this study is to analyse to what extent prescription patterns vary according to physician’s specialty, fragmentation of care and differences among public and private payer.

For this purpose, we consider a large longitudinal matched physician-prescription-patient dataset for all Portuguese e-prescriptions for the period between January 2015 and October 2019. We estimate the association between patient continuity of care mix and physician prescription rates for three pharmacotherapeutic groups – DPP-4i, SGLT2i and GLP-1 – across specialty types using a Negative Binomial regression approach.

Physicians vary their preferences towards prescription. Results suggest that Specialists play a secondary role on the prescription of DPP-4i and SGLT2i, while playing a central role on the prescription of GLP-1, in comparison with GPs.

More specifically, each specialist prescribes less 125 prescriptions in month $\left(n\right)$ when considering DPP-4i and 31.44 prescriptions when considering SGLT2i. For GLP-1, specialists add 44.25 prescriptions per month to their portfolio. Comparing with GPs and using a similar period of the considered dataset, 58 months, specialists would remove 7,250 and 1,821.2 prescriptions containing DPP-4i, SGLT2i, respectively. At the same time, they would add 2,566.5 prescriptions containing GLP-1.

This is due to the extensive presence of this condition on primary care setting due to disease management programs. Physicians are more likely to “prescribe new drugs in clinical and therapeutic areas where they feel familiar or have special interests” (Lublóy, 2014). Evidence suggests that GPs represent the largest category of clinicians prescribing glucose-lowering medications (Sangha et al., 2021) in comparison with Specialists (Jones et al., 2001) as they prescribe a considerable number of total prescriptions to a wide variety of patients, as opposed to medical specialists that normally treat an isolated population of patients (Nieuwenhuis, 2014; Alkabbani & Gamble 2023).

Recently, Alkabbani & Gamble (2023) confirmed that GPs and family physicians were responsible for most SGLT-2 inhibitor prescriptions. In fact, their study suggests that over 60% of all SGLT-2 inhibitor prescriptions were written by these physicians (Alkabbani & Gamble 2023).

Moretti et al., (2025), using a systematic review of physician prescription behaviour, found that primary care physicians (PCP) were found to be more likely to prescribe compared to specialists in 47% of included studies, and less likely in the remaining 23% (Moretti et al., 2025). Additionally, PCPs were found to be more likely to prescribe appropriately in 10% of the studies (Moretti et al., 2025).

When considering the fragmentation of care, results derived from the regression analysis considers that physicians with higher proportion of continuity arrangements present higher rates of prescription for all three pharmacotherapeutic groups.

In fact, physicians on single-pairing schemes prescribe approximately 60.32; 47.52 and 45.05 more prescriptions containing DPP-4i, SGLT2i and GLP-1, respectively. For a period of 58 months, this would translate into more 3,498.56; 2,756.16 and 2,612.9 prescriptions.

Continuity of care has been described as a three-layered concept that encompasses informational continuity, longitudinal continuity and interpersonal continuity. Interpersonal continuity is the most frequently examined aspect of continuity of care and it refers to the sustained and ongoing caring relationship, which catalyses the delivery of patient-centred healthcare, between the physician and the patient. It also reflects mutual trust and responsibility in this relationship (Chan et al., 2021).

Physicians are responsible for the current treatment as well as their maintenance. The agency relationship built from physician-patient repeated interactions is important since higher levels of continuity of care are able to reduce information asymmetry, benefit communication between the physician and the patient as well as it increases the physician’s knowledge on their patients’ disease history and current situation that might not be included in the patient’s medical records, social circumstances, values, and preferences (Chan et al., 2021; Culyer & Newhouse 2000; Saxell, 2014).

Knowledge and perceptions towards this interaction translate the quality of the interaction and the ability of physicians to learn from it and adjust their way of making further decisions.

The higher the number of interactions between the physician and patient the more feedback he gets as well as more diverse (Rourke et al., 2021). The circle of contacts becomes stronger, so they tend to receive more information and a constant update on knowledge level. Having the aspects of this relationship into account, it makes sense to assume that our results show that physicians are more likely to prescribe these hypoglycemic agents to patients that they see more often, suggesting that physicians are more open to prescription with patients that they know better.

Several literature on fragmentation of care can be found, especially if considering evidence from other health systems with similar GP-specialist structures. In 2017, the World Health Organization on its report “Medication Without Harm” considers multimorbidity, polypharmacy, and fragmented health care (i.e., patients attending appointments with multiple physicians in various health-care settings) as key drivers of medication-related harm, which may result in negative functional outcomes, high rates of hospitalisation, and excess morbidity and mortality, particularly in patients with frailty older than 75 years (WHO, 2024).

Prior et al., (2023), using data from Denmark, one of the closest structural comparators to Portugal (GP list system, strong primary care, tax-funded system), defended that care fragmentation was associated with higher rates of potentially inappropriate medication and increased mortality (Prior et al., 2023). Yet, frequent contact with the usual provider, fewer transitions, and better coordination were associated with better patient outcomes regardless of morbidity level (Prior et al., 2023). The study considers that even with a strong GP system, if patients’ care is spread across multiple providers and sectors without tight coordination, prescribing becomes more complex and less appropriate (Prior et al., 2023).

Using information from the UK health system, whose structure is also similar to Portugal (GP gatekeeping, registered lists, hospital specialists accessed through GP referral), Tarrant et al., (2023) consider that relational continuity, in the form of an ongoing GP-patient relationship over time, is increasingly difficult to achieve in the context of modern primary care (Tarrant et al., 2023). The authors defend the need for a single clinician with overall responsibility for medicines optimization (Tarrant et al., 2023).

The same standpoint is considered by Tsang et al., (2024). Using a UK-oriented work on polypharmacy and multimorbidity in primary care, they assume that in an organisational and systems level, fragmentation of care and poor coordination between healthcare teams and specialists often lead to deferring ownership of deprescribing, and miscommunication to patients, leading to medication-related problems (Tsang et al., 2024).

Also, lower levels of fragmentation in primary care as well as access regulation towards secondary care may result in lower prescribing drug costs (Lublóy et al., 2017; Frandsen et al., 2015; Bazemore et al., 2018; Kaltenborn et al., 2021).

The comparison of prescription trends amongst public and private payers suggests that an increase on the proportion of prescriptions with a public origin, decrease the prescription of DPP-4i, SGLT2i and GLP-1.

More specifically, a public setting would decrease 98.15 prescriptions in month $\left(n\right)$ when considering DPP-4i and 52.64 prescriptions when considering SGLT2i. For GLP-1, a public setting would present a higher expression with a decrease 97.38 prescriptions per month. Considering the same period of our dataset, 58 months, public setting would decrease the number of prescriptions in 5,693,7; 3,053,12 and 5,648,04 for DPP-4i, SGLT2i and GLP-1 respectively.

These are interesting findings as several health systems, including the Portuguese, that considers the duality between the public and private payer.

Different degrees of budget control are able to affect prescription behaviour (Lin et al., 2011).

Our results are aligned with Biglaiser & Ma (2007) and Lublóy (2014) that consider that physicians working in the private sector tend to prescribe more pharmaceuticals, than those who work in public workplaces (Lublóy, 2014; Biglaiser & Albert Ma 2007).

Regarding limitations, this study does not employ a formal causal identification strategy, and therefore the findings should be interpreted as associational rather than causal. The observed relationships may be influenced by potential confounders that were not fully accounted for, such as differences across socioeconomic status or access to healthcare services. In addition, the possibility of reverse causality cannot be excluded - for example in cases where physician prescription patterns arises when patient health status or outcomes influence prescribing behavior, rather than prescribing behavior influencing outcomes. This may lead to cases where physicians may prescribe more medications in response to worsening patient conditions, which can create the appearance that higher prescribing leads to poorer outcomes.

## Conclusion

This study analyses the extent to which prescription patterns vary according to physician specialty, fragmentation of care, and differences between public and private payers.

Results reflect that GPs represent the largest category of clinicians prescribing glucose-lowering medication. Also, physicians are more likely to prescribe hypoglycemic agents to patients that they see more frequently and that a higher proportion of prescriptions originating from the public sector is associated with a decrease in the use of DPP‑4i, SGLT2i, and GLP‑1 therapies.

This work contributes to the literature on fragmentation of care, physician specialty, and payer‑related differences in prescribing behaviour.

These insights are relevant both for the pharmaceutical industry—by informing how market strategies might be tailored to different prescriber profiles—and for policymakers seeking to understand how prescribing patterns influence patient outcomes and health system performance and sustainability.

While the study highlights potential implications for industry and health policy, these areas remain exploratory. Future work would benefit from translating these observations into concrete, actionable recommendations. For example, evaluating whether policies that strengthen continuity of care, such as reinforcing the role of the primary care physician as the central coordinator of chronic disease management, could promote more rational and consistent prescribing as well as improve medication adherence from a patient perspective. Similarly, examining how structured communication pathways between GPs and specialists, or targeted incentives for integrated care, might reduce fragmentation and support more appropriate adoption of innovative therapies would provide valuable guidance for decision-makers.

Overall, these findings open paths for further research on how physician specialty, care fragmentation, and payer origin shape the adoption of medicines, and how these dynamics ultimately affect therapeutic adherence and patient experience.
